# STAT3 inhibitor sensitized KRAS-mutant lung cancers to RAF inhibitor by activating MEK/ERK signaling pathway

**DOI:** 10.18632/aging.102244

**Published:** 2019-09-04

**Authors:** Zhenlin Wang, Mengchen Yin, Peilin Chu, Meiqing Lou

**Affiliations:** 1 Department of Neurosurgery, Shanghai General Hospital, Shanghai Jiao Tong University School of Medicine, Shanghai, China; 2 Department of Orthopaedics, LongHua Hospital, Shanghai University of Traditional Chinese Medicine, Shanghai, China; 3 Department of Orthopedics, The Central Hospital of Ma'anshan City, Anhui, China

**Keywords:** RAF, STAT3, AZ628, BP-1-102, lung cancer harboring KRAS mutations

## Abstract

KRAS is frequently mutated in patients with lung cancers, resulting in low survival rates. Inhibiting the downstream pathways of KRAS seems to be a feasible strategy to target KRAS-mutant tumors. However, the clinical outcomes only show limited success. Here, we developed a novel strategy by combining RAF (AZ628) and STAT3 (BP-1-102) inhibitors. The results showed that the AZ628 and BP-1-102 combination showed strongly synergistic effects on KRAS(G12D) H838, KRAS(G12S) H292 and KRAS(G12V) H441 cells and significantly enhanced the inhibition of cell proliferation *in vitro* and tumor growth *in vivo* by promoting apoptosis compared with one inhibitor alone. For mechanism, AZ628 and BP-1-102 combination markedly abrogated MEK/ERK signaling pathway activation in KRAS-mutant lung cancer cells suggesting the combination of RAF and STAT3 inhibitors is an effective therapy for treating lung cancer cells harboring KRAS mutations. Taken together, the current results indicate that oncogene addiction can be targeted for therapy in lung cancer cells harboring RAS-mutant.

## INTRODUCTION

Oncogenic mutant RAS proteins (KRAS, NRAS and HRAS) are prevalent in up to 30% of all human cancers [1]. In mammalian cells, the RAS genes function as molecular switches that regulate cell growth, differentiation and survival [2]. As a major isoform of RAS, it is frequently mutated in multiple cancers, such as pancreatic, colorectal and lung cancers. In lung adenocarcinomas, it is estimated that 25%–30% of patients harbor activating KRAS mutations, which have lower survival rates than those without KRAS mutations [3, 4]. KRAS mutant lung adenocarcinoma cancer shows a poor prognosis compared with other solid tumors and molecular subtypes of lung cancer due in part to the progress of resistance to currently systemic or targeted therapy [5]. Accordingly, it’s urgent to develop novel or improved therapies for the treatment of this particular type of lung malignancy.

It seems that inhibiting KRAS mutant directly is a promising concept for novel treatment strategy according to the effective treatment of EGFR inhibitors for EGFR mutant tumors. However, direct pharmacologic targeting of activated RAS protein with small molecules is difficult because of the deficiency of suitable small-molecular binding sites [6]. The downstream effectors of KRAS signals include MAPK and STAT3 signaling cascade [7]. Among them, MAPK signaling plays an important role in KRAS mutant lung cancer, and the components of this pathway have been widely used to develop drugs for KRAS inhibition. The mitogen-activated protein kinase (MAPK) pathway is also known as RAS/RAF/MEK/ERK pathway, regulating cell proliferation, differentiation, apoptosis and survival [8]. This signaling cascade is triggered by the activation of RAS G protein, further activating RAF proto-oncogene serine/threonine-protein kinase, resulting in mitogen-activated protein kinase kinase (MEK) phosphorylation [9]. Subsequently, extracellular signal-regulated kinase (ERK) is activated, leading to cell migration, proliferation and survival [10]. It is known that MEK inhibitors, such as AZD6244 and trametinib, have been developed in clinical studies in cancer, and have the potential to suppress KRAS mutant tumor rely on MAPK signaling pathway [7]. However, drug resistance to MEK inhibitors has been reported in cancer with KRAS mutations [11, 12].

STAT3, an oncogene that plays an important role in cell proliferation, survival, angiogenesis and immunosuppression, has been identified as an essential molecule in RAS oncogenic transformation [13, 14]. The activation of STAT3 induces oncogenic transformation in cells and tumor formation in nude mice [7]. In addition, STAT3 is also reported as a marker of poor prognosis [15]. Previous studies have reported that STAT3 signaling pathway is aberrantly activated in the development of KRAS mutant lung tumors [16] and the deficiency of STAT3 inhibits KRAS mutant-driven lung tumorigenesis in female mice [5]. As such, STAT3 is an attractive target for KRAS mutant tumor treatment.

It is well known that MEK inhibitors induce STAT3 phosphorylation/activation. Since MEK inhibitors have met with limited clinical success in single-agent therapy, the combination of MEK inhibitor and STAT3 pathway modulator has arouse interest of the researchers. Zhao et al. reports that the activation of STAT3 may be the mechanism for resistance to MEK inhibitor, and the combination of STAT3 and MEK inhibitors can be used as potential therapy for pancreatic and colon cancers with KRAS mutant [7]. Yoon et al. shows that combined STAT3 inhibition with MEK inhibition restrains STAT3 activation, resulting in synergistical suppression of cell growth and stimulation of apoptosis in KRAS mutant lung cancer cells [3].

In this study, we characterized the combination therapy of AZ628, the inhibitor of RAF, and BP-1-102, the inhibitor of STAT3, in KRAS mutant lung cancer. The combination showed a strongly synergistic interaction in KRAS-mutant lung cancer cells compared with wild-type cancer cells, and KRAS-mutant lung cancer cells were more sensitive to this combination than AZ628 or BP-1-102 single. Clone formation assay *in vitro* and xenograft mice models *in vivo* confirmed this finding. In addition, though this combination or single agent significantly caused the apoptosis of both KRAS mutant cancer cells and wild-type cells, it is observed that the two inhibitors’ combination obviously enhanced the ability of apoptosis induction in lung cancer cells harboring KRAS mutation, indicating the combination of RAF and STAT3 inhibitors is an effective therapy for treating lung cancer cells harboring KRAS mutations.

## RESULTS

### KRAS-mutant lung cancer cells are selectively sensitive to the combined inhibition of RAF and STAT3

**Figure 1:**
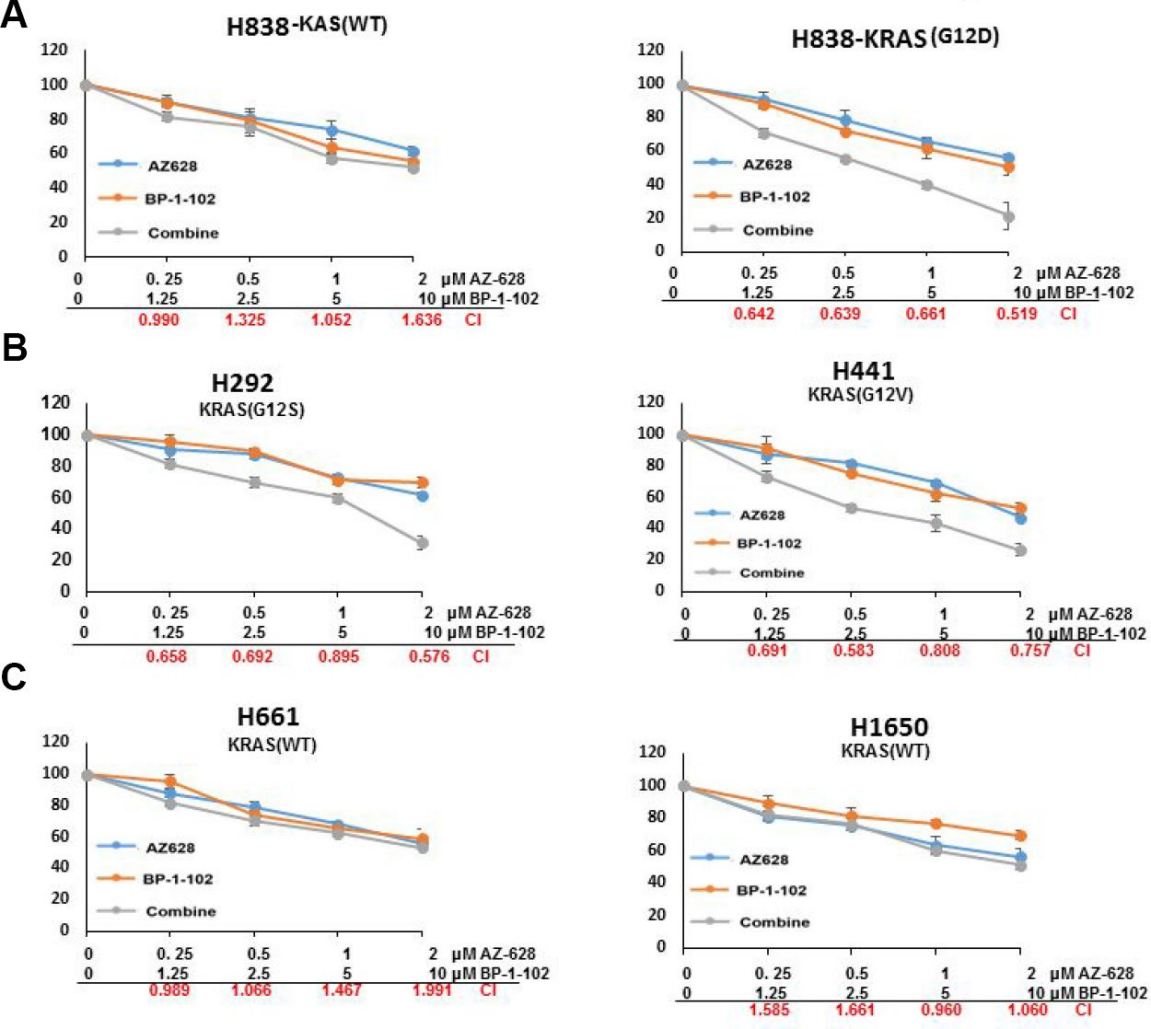
**The combination of AZ628 and BP-1-102 showed a strongly synergistic interaction in KRAS-mutant lung cancer cells.** Relative viability was measured for KRAS(WT) H838 and KRAS(G12D) H838 cells (**A**), KRAS(G12S) H292 and KRAS(G12V) H441 cells (**B**), as well as KRAS(WT) H661and H1650 cells (**C**) that were treated with the single drug or combined drugs. CI values were calculated for cells treated with a combination of AZ 628 and BP-1-102. CI < 0.7 is considered as synergism; CI = 0.7–0.9 is moderate synergism; CI = 0.90–1.10 is nearly additive; and CI > 1.10 is antagonism.

AZ628 is one of the inhibitors of RAF, and BP-1-102 is a STAT3 inhibitor. To evaluate the therapeutic effect of combined AZ628 and BP-1-102 on lung cancer cells, we analyzed the interaction (synergistic, additive or antagonistic) by calculating the combination index (CI). CI < 0.7 is considered as synergism; CI = 0.7–0.9 is moderate synergism; CI = 0.90–1.10 is nearly additive; and CI > 1.10 is antagonism. The cytotoxicity of combined AZ628 and BP-1-102 is enhanced in KRAS(G12D) H838 cells compared with KRAS(WT) H838 cells, and the CI values were < 0.7 in all groups with different concentrations combination, suggesting a strongly synergistic interaction between AZ628 and BP-1-102 in KRAS mutant lung cancer cells (Figure 1). In KRAS(G12S) H292 and KRAS(G12V) H441 cells which had KRAS mutation, the combination showed obvious synergism in some concentrations, whereas in other concentrations the drug effect was nearly additive. In contrast, antagonistic interaction between this combination drugs was observed in H661 and H1650 cells which were KRAS wild-type lung cancer cells. These findings indicated that KRAS mutant lung cancer cells might be selectively sensitive to combined AZ628 and BP-1-102.

### The combination of RAF and STAT3 inhibitors enhanced the inhibition of KRAS mutant lung cancer cells growth

**Figure 2:**
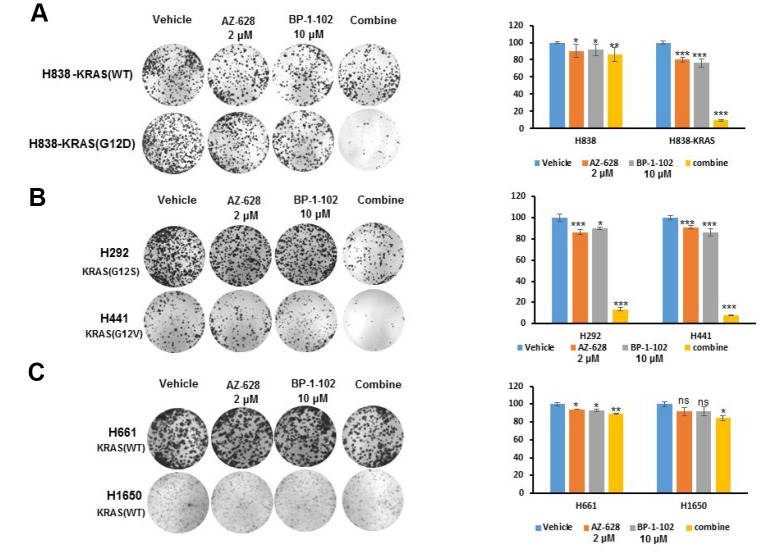
**The combination of AZ628 and BP-1-102 enhanced the inhibition of KRAS mutant lung cancer cells growth.** (**A**) Clonogenic assay was performed for KRAS(WT) H838 and KRAS(G12D) H838 cells treated with single or combination drugs. The statistical analysis was also demonstrated. * p<0.05. **p<0.01. ***p<0.001. (**B**) Clonogenic assay was performed for KRAS(G12S) H292 and KRAS(G12V) H441 cells treated with single or combination drugs. The statistical analysis was also tested. * p<0.05. ***p<0.001. (**C**) Clonogenic assay was performed for KRAS(WT) H661and H1650 cells treated with single or combination drugs. The statistical analysis was also addressed. * p<0.05. **p<0.01.

To confirm the synergistic effect of RAF and STAT3 inhibitors, we evaluated their roles in the growth of KRAS mutant lung cancer cells by using AZ628 (2 μM) and BP-1-102 (10 μM). The clonogenic assays revealed that in KRAS(G12D) H838 cells, this combination caused more enhanced inhibition effect on cell growth than either agent alone, whereas there is no significant difference in H838 cell growth (Figure 2A). The similarly enhanced inhibition effects of this combination were also observed in KRAS(G12V) H441 and KRAS(G12S) H292 cells (Figure 2B). In contrast, in KRAS(WT) H661and H1650 cells, the results showed no significant effect of combined AZ628 and BP-1-102 on cell growth (Figure 2C).

### The combination of RAF and STAT3 inhibitors induced cell apoptosis increase

**Figure 3:**
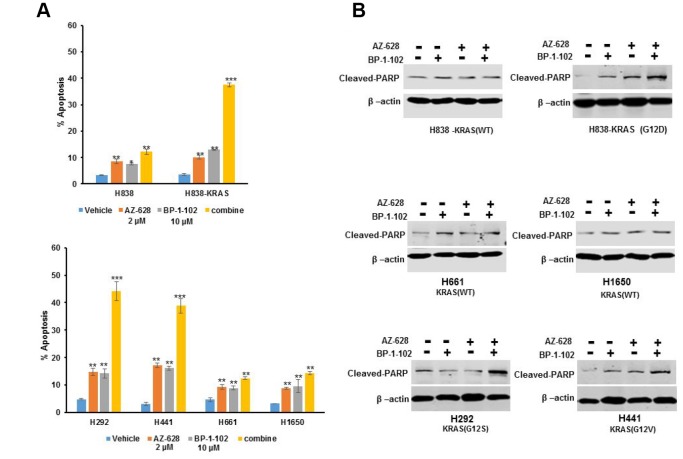
**The combination of AZ628 and BP-1-102 induced cell apoptosis increase.** (**A**) Flow cytometry was performed to detect cell apoptosis induced by single or combination drugs in KRAS(G12D) H838, KRAS(G12S) H292, KRAS(G12V) H441, KRAS(WT) H838, H661and H1650 cells. * p<0.05. **p<0.01. ***p<0.001. (**B**) Western blot was performed to detect cleaved PRAP protein levels caused by single or combination drugs in different cells.

Cell apoptosis assays were performed with single inhibitor or a combination of AZ628 and BP-1-102 on a panel of KRAS mutant lung cancer cells and wild-type lung cancer cells. The results showed that single inhibitor also owned the ability to induce cell apoptosis in both KRAS mutant lung cancer cells and wild-type lung cancer cells (Figure 3A). However, KRAS(G12D) H838, KRAS(G12V) H441 and KRAS(G12S) H292cells showed far higher apoptotic response to the combination than to single inhibitor compared with KRAS(WT) H838, H661and H1650 cells.

PolyADP-ribose polymerase (PARP) is involved in repair of damages to single-strand DNA, and plays a critical role in cell apoptosis [17]. Thus, we further performed cleaved PARP immunoblot assay to investigate whether apoptosis induction caused growth inhibition in a panel of KRAS mutant lung cancer cells. Either no or minimal effects on cleaved PARP expression were observed in KRAS(G12D) H838, KRAS(G12V) H441 and KRAS(G12S) H292 treated with AZ628 or BP-1-102 alone, whereas the combination induced a significant increase of cleaved PARP production (Figure 3B). In addition, there is no significant effects of the combined inhibitors on cleaved PARP expression in KRAS(WT) H838, H661and H1650 cells.

### Combined inhibition of RAF and STAT3 blocked MEK/ERK signaling pathway

**Figure 4:**
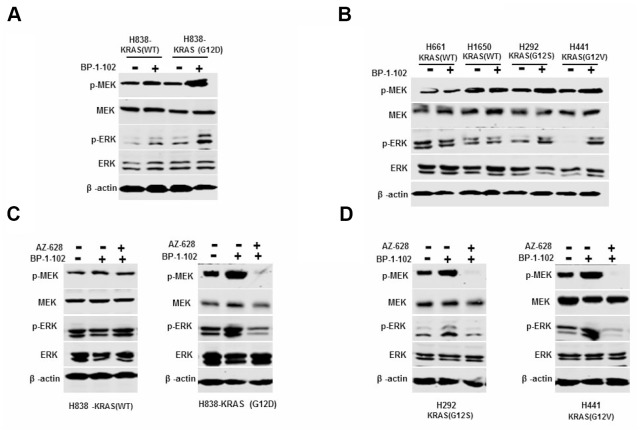
**Combined AZ628 and BP-1-102 blocked MEK/ERK signaling pathway.** (**A**) Western blot determined the protein levels of p-MEK and p-ERK in H838 and KRAS(G12D) cells treated with BP-1-102 alone. (**B**) Western blot determined the protein levels of p-MEK and p-ERK in KRAS(G12S) H292 and KRAS(G12V) H441 cells, as well as KRAS(WT) H661and H1650 cells treated with BP-1-102 alone. (**C**) Western blot determined the protein levels of p-MEK and p-ERK in KRAS(WT) H838 and KRAS(G12D) H838 cells treated with AZ628 and BP-1-102combination drugs. (**D**) Western blot determined the protein levels of p-MEK and p-ERK in KRAS(G12S) H292 and KRAS(G12V) H441 cells treated with AZ628 and BP-1-102combination drugs.

To determine whether the synergistic effects on KRAS mutant lung cancer cells were associated with MAPK pathway, we treated a panel of KRAS(G12D) H838, KRAS(G12V) H441 and KRAS(G12S) H292cells and KRAS(WT) H838, H661and H1650 cells with AZ628 and BP-1-102 combination or BP-1-102 alone. The western blot assay showed that BP-1-102 alone increased the protein levels of p-MEK and p-ERK in KRAS mutant lung cancer cells compared with cells without treatment, whereas in wild-type cells, BP-1-102 alone had little effect on their protein levels (Figure 4A, 4B). Interestingly, the expression levels of p-MEK and p-ERK were significantly inhibited in KRAS(G12D) H838, KRAS(G12V) H441 and KRAS(G12S) H292 cells treated with AZ628 and BP-1-102 combination relative to KRAS mutant cells without treatment (Figure 4C, 4D). These findings suggested that STAT3 inhibitor single stimulated MEK/ERK signaling pathway activity, whereas the combination of RAF and STAT3 inhibitors significantly suppressed this pathway.

### Combined RAF and STAT3 inhibitors enhanced therapeutic response *in vivo*

**Figure 5:**
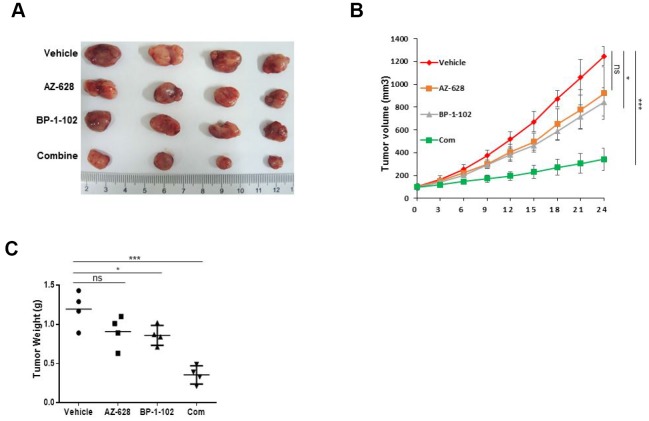
**Combined AZ628 and BP-1-102 inhibited tumor growth *in vivo*.** (**A**) Photographs of the xenograft KRAS(G12V) H441 tumors treated with single or combination drugs. (**B**) Tumor volume was measured. (**C**) Tumor weight was measured. *p<0.05. ***p<0.001.

Given that the efficacy of combined RAF and STAT3 inhibitors against KRAS mutant cells *in vitro*, this study further evaluated their therapeutic efficacy *in vivo*. A xenograft mice model of lung cancer harboring KRAS mutant was established by using KRAS(G12V) H441 cells. The results showed that BP-1-102 alone significantly decreased the tumor volume and weight, whereas tumor growth in mice treated with AZ-628 alone was not significant different from mice without treatment (Figure 5). However, the combination resulted in an enhanced tumor growth inhibitory effect that both tumor volume and weight were remarkably reduced in mice treated with the combined drugs compared with vehicle and BP-1-102 alone.

## DISCUSSION

RAF includes A-RAF, b-RAF and c-RAF/RAF-1, which is involved in MAPK signaling cascade [18]. RAF-1 regulates the activation of anti-apoptotic transcription factor, nuclear factor-κB, and caspase-8 [19]. B-RAF is frequently mutated in human tumors, further stimulating cancer cell growth and survival via improving kinase activity and inducing the downstream MEK-ERK pathway [20]. It is also reported that b-RAF mutation is one of the most frequent cause of MAPK pathway aberrant activation [21]. Thus, inhibiting activation of RAF may be a crucial treatment for preventing cancer cell growth. However, previous study has demonstrated that RAF inhibitors show little efficacy in KRAS mutant cancers [22], which suggests that tumors harboring KRAS mutations may be insensitive to single-agent RAF inhibitions. In this study, we found that RAF inhibitor (AZ628) combined with STAT3 inhibitor (BP-1-102) reversed this little efficacy, and had a great effect on suppressing the growth of KRAS mutant cancer cells and tumor.

AZ628, a type II selective pan-RAF kinase inhibitor, inhibits the activity of preactivated b-RAF, b-RAF V600E and c-RAF, blocking MEK activation and sustaining sensitivity to the MEK inhibitor, thus resulting in cancer cell proliferation inhibition [19, 23, 24]. BP-1-102, one of the STAT3 inhibitors, blocks STAT3 phosphorylation at tyrosine 705 (Tyr705), and further inhibits STAT3 dimerization, DNA binding, and downstream genes activation [25]. This study found that the combination of AZ628 and BP-1-102 showed a strongly synergistic interaction in KRAS-mutant lung cancer cells compared with wild-type cancer cells, and KRAS-mutant lung cancer cells were more sensitive to this combination than AZ628 or BP-1-102 single. Clone formation assay *in vitro* and xenograft mice models *in vivo* confirmed this finding. AZ628 and BP-1-102 combination significantly inhibited the growth of KRAS-mutant cancer cells and tumors, whereas single agent only had slight inhibition ability, which is consistent to the report of previous study that RAF inhibitors show little efficacy in KRAS mutant cancers [22].

We further determined the role of combined inhibitors in cell apoptosis. Though this combination or single agent significantly caused the apoptosis of both KRAS mutant cancer cells and wild-type cells, it is observed that the two inhibitor’s combination obviously enhanced the ability of apoptosis induction in lung cancer cells harboring KRAS mutation. Cleaved PARP immunoblot assay revealed that treatment with AZ628 or BP-1-102 single selectively increased cleaved PARP protein levels in KRAS mutant cancer cells, while these inhibitors combination significantly induced cleaved PARP expression in these cells. Cleaved PARP is mainly involved in repair of damages to single-strand DNA, and plays a critical role in cell apoptosis [17]. This perfectly explains that the inhibition of cell growth caused by AZ628 and BP-1-102 combination is due to the increase of cell apoptosis.

To investigate the mechanism underlying the effects of combined AZ628 and BP-1-102 on KRAS mutant lung cancer cells, we evaluated the role of these inhibitors in MEK/ERK signaling pathway. STAT3 has been reported to be an essential molecule in RAS oncogenic transformation [13]. This study showed that BP-1-102 markedly increased the protein levels of p-MEK and p-ERK in KRAS mutant cancer cells, suggesting that STAT3 inhibitor can result in MEK and ERK up-regulation. Interestingly, p-MEK and p-ERK expression were significantly inhibited when BP-1-102 combined with AZ628. Given the distinct pharmacological targeting profiles of AZ628 and BP-1-102, future work should also address how to impose the pharmacological effects of these two drugs in RAS-mutant cells?

**Figure 6:**
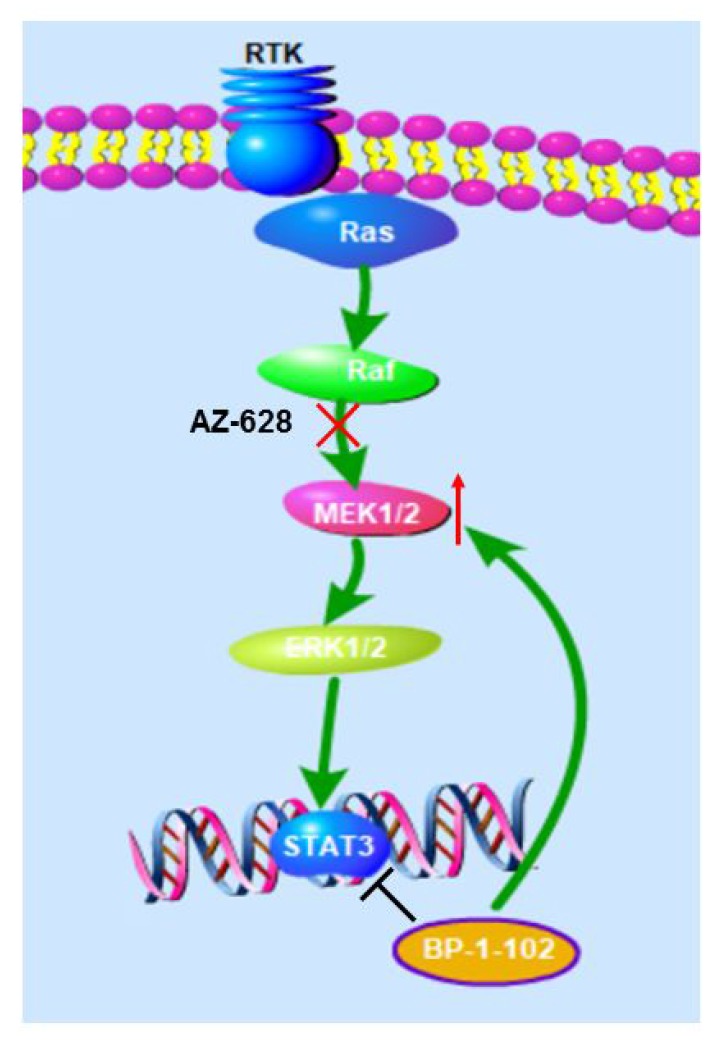
**Proposed model.** Pictures showing that AZ628 and BP-1-102 combination inhibits RAS-mutant lung cancer cells by markedly abrogating MEK/ERK signaling pathway activation.

In summary, the current data support the pharmacological action mode that AZ628 and BP-1-102 combination inhibits RAS-mutant lung cancer cells by markedly abrogating MEK/ERK signaling pathway activation (Figure 6). Our new findings on the pharmacological effects of AZ628 and BP-1-102 combination on lung cancer cells may pave a new way to treat this refractory cancer.

## MATERIALS AND METHODS

### Cell culture and reagents

Human lung cancer cell lines, including H838, H292, H441, H661, and H1650, were purchased from the American Type Culture Collection (ATCC; Manassas, USA), and cultured in RPMI-1640 or DMEM supplemented with 10% fetal bovine serum (FBS; Gibco, Gaithersburg, MD, USA) at 37°C in a humidified incubator filled with 5% CO_2_. AZ628, a RAF inhibitor, and BP-1-102, a STAT3 inhibitor, were obtained from Medchemexpress. Stock solutions were prepared in DMSO and stored at −20°C.

### Cell viability assay

Cell Counting Kit-8, a variant of MTT assay, was performed to detect the cytotoxic effects of AZ628 or BP-1-102 or their combination on lung cancer cells. Cells were transformed to 96-well plates at a density of 3000 cells/well, and incubated for 24 h. Subsequently, cells were treated with different concentrations of AZ628 or BP-1-102 or DMSO as control. Cell viability was measured after 72 h. Combination index (CI) was calculated by CalcuSyn software. CI < 0.7 is considered as synergism; CI = 0.7–0.9 is moderate synergism; CI = 0.90–1.10 is nearly additive; and CI > 1.10 is antagonism. All reactions were performed in triplicate.

### Clonogenic assay

Cells were plated in 6-well plates and continuously incubated for 24 h. And then, cells were treated with DMSO or 2 μM AZ628 or 10 μM BP-1-102 or a combination of AZ628 and BP-1-102 for 7 days, and stained with crystal violet.

### Flow cytometry assay

After treated with drugs for 72 h, cells were harvested, and stained by PI (100 μg/ml) and Annexin V-Alexa Fluor488 conjugate at room temperature for 20 min in dark. And then, cell apoptosis was detected by using flow cytometer (BD Biosciences, Franklin Lakes, NJ). The extent of apoptosis was quantified as a percentage of annexin V FITC-positive cells.

### Western blot assay

Cells were treated with DMSO or 2 μM AZ628 or 10 μM BP-1-102 or a combination of AZ628 and BP-1-102 for 24 h. Proteins were extracted by using RIPA lysis buffer, and then separated by 10% SDS-PAGE gels, following by transferring to polyvinylidene difluoride (PVDF) membrane. After blocking in 5% skim milk at room temperature, the membranes were probed with primary antibodies overnight at 4°C. β-actin was used as an endogenous loading control. Subsequently, the membranes were incubated with diluted goat polyclonal anti-rabbit IgG secondary antibody (1:2000; Abcam). An enhanced chemiluminescence system was further performed to detect protein levels.

### Xenograft study

BALB/c nude Mice, 6–8 week old, were purchased from the SLAC Laboratory Animal Center (Shanghai, China), and were subcutaneously injected with H441 cells (5×10^6^) in the right flank. And then, mice were divided into four groups treated with vehicle, AZ628, BP-1-102, and in combination, respectively. AZ628 was dosed at 20 mg/kg and BP-1-102 at 20 mg/kg. And then, mice were sacrificed. Tumor volume was detected by using a digital caliper.

### Statistical analysis

All experiments were performed at least three times. Data are presented as mean ± SEM. Statistics analysis was performed with SPSS software. Student’s t-test was used to determine the significance between the tested groups. *P*<0.05 was regarded statistically significant difference.
